# Single-strain behavior predicts responses to environmental pH and osmolality in the gut microbiota

**DOI:** 10.1128/mbio.00753-23

**Published:** 2023-07-11

**Authors:** Katharine M. Ng, Sagar Pannu, Sijie Liu, Juan C. Burckhardt, Thad Hughes, Will Van Treuren, Jen Nguyen, Kisa Naqvi, Bachviet Nguyen, Charlotte A. Clayton, Deanna M. Pepin, Samuel R. Collins, Carolina Tropini

**Affiliations:** 1 Department of Microbiology and Immunology, University of British Columbia, Vancouver, Canada; 2 School of Biomedical Engineering, University of British Columbia, Vancouver, Canada; 3 Independent Researcher, Vancouver, British Columbia, Canada; 4 Department of Microbiology and Immunology, Stanford University School of Medicine, Stanford, California, USA; 5 Humans and the Microbiome Program, Canadian Institute for Advanced Research, Toronto, Canada; Rutgers University, Piscataway, New Jersey, USA

**Keywords:** acid stress, machine learning, microbiota, osmolality, single-strain culture, culturomics

## Abstract

Changes to gut environmental factors such as pH and osmolality due to disease or drugs correlate with major shifts in microbiome composition; however, we currently cannot predict which species can tolerate such changes or how the community will be affected. Here, we assessed the growth of 92 representative human gut bacterial strains spanning 28 families across multiple pH values and osmolalities *in vitro*. The ability to grow in extreme pH or osmolality conditions correlated with the availability of known stress response genes in many cases, but not all, indicating that novel pathways may participate in protecting against acid or osmotic stresses. Machine learning analysis uncovered genes or subsystems that are predictive of differential tolerance in either acid or osmotic stress. For osmotic stress, we corroborated the increased abundance of these genes *in vivo* during osmotic perturbation. The growth of specific taxa in limiting conditions in isolation *in vitro* correlated with survival in complex communities *in vitro* and in an *in vivo* mouse model of diet-induced intestinal acidification. Our data show that *in vitro* stress tolerance results are generalizable and that physical parameters may supersede interspecies interactions in determining the relative abundance of community members. This study provides insight into the ability of the microbiota to respond to common perturbations that may be encountered in the gut and provides a list of genes that correlate with increased ability to survive in these conditions.

**IMPORTANCE:**

To achieve greater predictability in microbiota studies, it is crucial to consider physical environmental factors such as pH and particle concentration, as they play a pivotal role in influencing bacterial function and survival. For example, pH is significantly altered in various diseases, including cancers, inflammatory bowel disease, as well in the case of over-the-counter drug use. Additionally, conditions like malabsorption can affect particle concentration. In our study, we investigate how changes in environmental pH and osmolality can serve as predictive indicators of bacterial growth and abundance. Our research provides a comprehensive resource for anticipating shifts in microbial composition and gene abundance during complex perturbations. Moreover, our findings underscore the significance of the physical environment as a major driver of bacterial composition. Finally, this work emphasizes the necessity of incorporating physical measurements into animal and clinical studies to better understand the factors influencing shifts in microbiota abundance.

## INTRODUCTION

The animal digestive tract naturally consists of numerous distinct environments in which physical and chemical conditions such as oxygen concentration, acidity, mucosal stiffness, and temperature are tightly regulated by host–microbial interactions. Environmental gradients along the intestine create a continuum of habitats that the microbiota explores in its voyage along the intestinal tract (1 - 3). Alterations in pH and particle concentration (osmolality) commonly occur in gut disease or result from the ingestion of specific compounds. For example, inflammatory bowel disease (IBD), intestinal cancers, and antacids are associated with abnormal pH values (4, 5). Furthermore, diarrhea and aging are associated with increased oxygen in the intestine, and conditions such as malabsorption due to intolerances (e.g., celiac disease) or overconsumption of salts, alcohol, and laxatives lead to changes in osmolality (6 - 8). Additionally, the microbiota affects physical parameters by consuming luminal oxygen, degrading the mucosal layer, or acidifying the environment through fermentation and short-chain fatty acid (SCFA) production (9 - 11).

Changes in the gut physical environment affect the gut microbiota on a broad scale, favoring growth only when the biochemical and physical conditions match the requirements of each taxon (3). The survival of specific taxa is driven by the function of genes and pathways that regulate metabolism and stress responses over short and chronic time scales. These genes and pathways play a key role in establishing the microbiota members that can grow in specific regions of the gut and host states (1 - 3). For example, the steep intraluminal oxygen gradient partitions strictly anaerobic bacteria such as *Faecalibacterium* away from the more oxygenated epithelium, while more aerotolerant bacteria such as Enterobacteriaceae can associate with the mucosa (12). Beyond oxygen sensitivity, pH and osmolality also impact bacterial growth and survival (4, 13 - 15). Even small alterations in pH and osmolality can dramatically affect bacterial growth due to alterations in enzyme activity, energetic favorability of certain nutrient substrates, and rates of protein synthesis (16 - 18).

Previous studies have highlighted broad differences among microbial taxa in their adaptation to pH and osmolality, as evidenced by the differential enrichment of well-studied taxa in response to pH alterations (13). For example, *Lactobacillus* species propagate over wide ranges of pH, whereas acidic environments inhibit some members of the *Bacteroides* genus (19). Despite these general trends, certain members within these taxonomic groups excel in high-stress conditions of changed pH and osmolality, while others display sensitivity to these parameters, indicating genus- or even strain-specific differences (4, 20). Even in the absence of limiting cases in which taxa cannot grow, changing physical conditions can affect bacterial growth rates, resulting in microbiota composition shifts within the highly competitive and nutrient-depleted environment of the intestine. For example, mild osmotic diarrhea induced by polyethylene glycol (PEG) can induce long-term changes in gut microbial membership despite presenting no change in bacterial density or load (14).

Thus far, the effects of physical parameters on bacterial growth across intestinal bacterial taxa have not been well documented. Importantly, the taxonomic level at which growth phenotypes can be generalized remains unclear; moreover, it remains unknown whether the physical environment is broadly predictive of bacterial response and abundance. Closing this gap of understanding is particularly critical for identifying the relationship between the microbiota and disease, as the presence of certain gut bacterial members may be strongly dictated by the physical environment rather than disease-specific phenotypes. In addition, identifying the genes that allow particular gut members to survive varying physical parameters is crucial. This knowledge would also shed light on the effectiveness of microbiota therapies, as procedures such as fecal microbiota transplant or probiotic administration may be rendered ineffective by the transfer of members that cannot survive in the disease-altered environment.

In this work, we examined the growth phenotypes of 92 species from 28 families across a range of pH and osmolality values. We combined high-throughput growth measurements, environmental measurements, and machine learning (ML)-assisted comparative genomics to systematically identify the capacity of microbial taxa to survive in pH and osmolality conditions relevant to health and disease. We performed a thorough *in vitro* analysis of bacterial growth of individually grown strains and revealed general trends of tolerance among phylogenetically related microbes, including known pathogens and probiotics strains. We corroborated these results by performing human microbiota community experiments *in vitro* and in humanized mouse models and demonstrated the broad predictability of bacterial abundance across multiple donors and conditions. Importantly, we found that *in vitro* results of single-strain stress tolerance can predict bacterial behavior in complex *in vivo* conditions. We also found that the presence of genes involved in osmotic stress response is predictive of survival in an environment with disrupted osmolality. Taken together, our results demonstrate that the physical environment is broadly predictive of bacterial response and abundance. This knowledge will aid in determining the effectiveness of microbiota therapies and in assessing whether treatments may be viable in a given perturbed gut environment.

## RESULTS

### Collection of 92 strains from 28 families of bacteria

**Fig 1:**
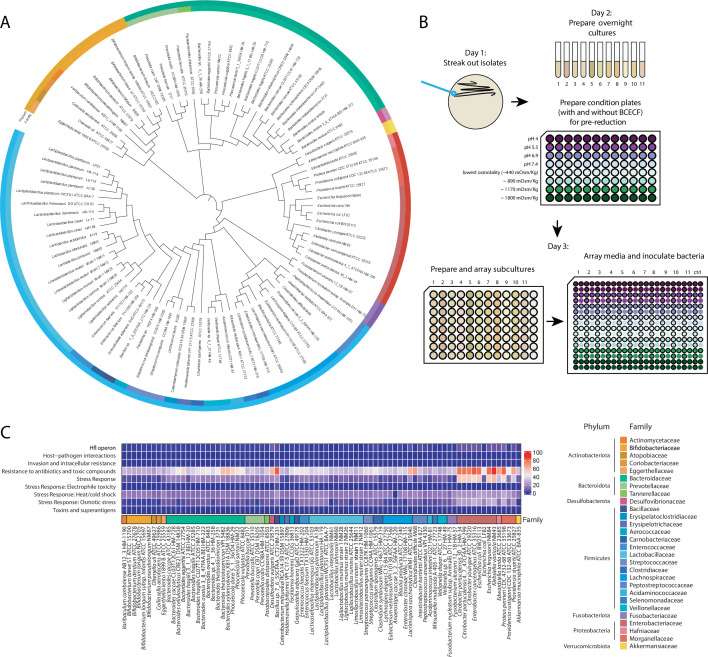
Phylogenetic overview and experimental setup of characterized intestinal bacterial strains. (**A**) 16S rRNA sequences from each member of the strain library were acquired from the SILVA database and used to generate a phylogenetic tree. (**B**) Experimental design and workflow for the characterization of growth of bacterial strains under different physical conditions. (**C**) Heatmap of PATRIC annotations of characterized strains within the subcategories of the Stress Response, Defense, and Virulence gene categories.

We cultured 92 bacterial strains from 28 common gut bacterial families across seven phyla, comprising a diverse set of strains with a focus on human isolates (Fig. 1A). We chose these strains based on their public availability, fully sequenced genomes, and broad interest due to their prevalence in the gut microbiota. We derived most strains from the BEI collection of Human Microbiome Project human strains, the American Type Culture Collection (ATCC), the Deutsche Sammlung von Mikroorganismen und Zellkulturen (DSMZ), and the Collection of Inflammation-Associated Mouse Intestinal Bacteria (21) (Materials and Methods). Of these strains, 69 were human isolates, 10 were mouse-derived, and the remaining 13 were either isolated from probiotics or other strain types. Some characterized probiotic strains (11) were isolated from commercial sources and were not previously sequenced. For taxa of high interest due to their prevalence, abundance, or health relevance, we included multiple species or strains within a family in order to avoid drawing species-specific conclusions (22 - 30). Specifically, we increased the coverage of the Bacteroidaceae*,* Bifidobacteriaceae*,* Lactobacillaceae*,* Lachnospiraceae*,* Enterobacteriaceae*,* and Prevotellaceae families. To facilitate high-throughput cultivation and comparisons of strains, we grew the majority of the bacteria (83/92) in anaerobic conditions in Mega Medium, a rich and undefined medium previously demonstrated to support the growth of a wide variety of strains (Materials and Methods) (31). The remaining strains required more specialized media for growth (Materials and Methods; Fig. 1B; Table S1). To measure the impact of bacterial growth on the environmental pH, we supplemented the experimental media with 2'′,7′-bis-(2-carboxyethyl)-5-(and-6)-carboxyfluorescein (BCECF), which enabled real-time pH measurements coupled with optical density (OD) measurements (Fig. 1B). We uncovered no significant trends in medium acidification with respect to growth in different conditions (Fig. S1A). As the genomes in our strain library have been fully sequenced and assembled, we performed comparative genomics analyses that combined protein annotations from the Pathosystems Resource Integration Center (PATRIC). To visualize this analysis across all strains, we created a novel visualization tool to explore PATRIC annotation data and compare across multiple genomes (https://tropinilab.shinyapps.io/strain_heatmap_app/) (Fig. 1C). We grew the strains in eight different conditions, spanning four osmolalities (from unmodified medium osmolalities of 0.23–0.44 Osm/kg to a maximum condition of 1.8 Osm/kg) and four pH values (4–7.4). We selected these ranges to mimic the potential environmental conditions that intestinal bacteria encounter along the gastrointestinal tract and during perturbations (14, 32, 33).

### Bacterial families display a range of tolerance to increasing osmolality

**Fig 2:**
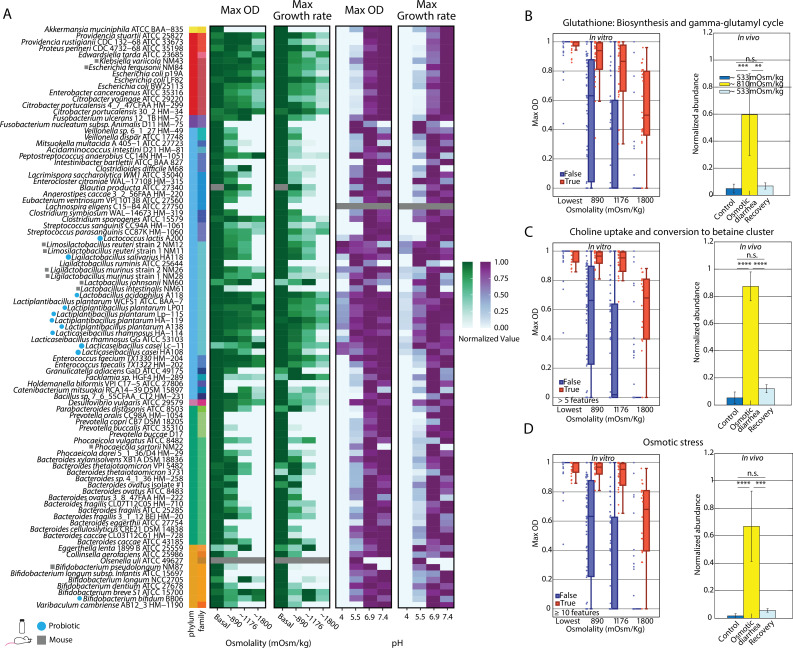
Osmotic and pH stress responses lead to phenotype variations. (**A**) Heatmap displaying normalized growth rate and OD_600_ of 92 characterized bacterial strains in osmotic conditions ordered by phylogenetic relatedness. The growth rates and OD were normalized to maximum growth rate and OD values within a characterized strain across osmolality and pH conditions. The growth curves characterized in conditions of varying osmolality and pH demonstrate general trends of tolerance across bacterial taxa. (B–D) ML analysis of PATRIC annotations and growth profile data of characterized strains obtained via a one-level decision tree regression model. (**B**) Boxplot of the presence (red) or absence (blue) of bacterial species possessing features in glutathione biosynthesis and the gamma-glutamyl cycle as a function of the maximum OD across osmolality conditions of ~440, 890, 1,176, and 1,800 mOsm/kg (left). This pathway is found to be enriched *in vivo* during osmotic perturbation (right). (**C**) Boxplot of characterized bacterial species encoding (red) or missing (blue) a gene for the choline uptake and conversion to betaine cluster as a function of the maximum OD under varying osmolality conditions (left). This pathway is found to be enriched *in vivo* during osmotic perturbation (right). (**D**) Boxplot highlighting the presence (red) or absence (blue) of at least 10 osmotic stress genes as a function of the maximum OD in varying osmotic conditions (left). This pathway is found to be enriched *in vivo* during osmotic perturbation (right). **P < 0.01, ***P < 0.001, ****P < 0.0001.

Increasing osmolality elicited widely divergent effects on the bacterial families assayed in this study (Fig. 2A). While *in vivo* measurements of high gut osmolality are usually less than ~1,100 mOsm/kg (14), we sought to explore a wider range of high osmolalities, as many bacterial taxa showed strong growth at these values in the current study. Lactobacillaceae and Enterobacteriaceae family members displayed robust growth at moderately high (~1,176 mOsm/kg) and high (~1,800 mOsm/kg) osmolalities. Interestingly, we observed modest heterogeneity among genera and species within Lactobacillaceae; the strains that were more negatively affected in high-osmolality conditions were *Lactobacillus murinus* strains 1 (NM26) and 2 (NM28), *Lactobacillus intestinalis* NM61, and *Lacticaseibacillus rhamnosus* HA-114. Conversely, bacteria in the Bacteroidaceae, Bifidobacteriaceae, and Lachnospiraceae families displayed a wide range of sensitivities to increasing osmolality; except for two *Bifidobacterium* strains, these bacteria were unable to grow in media with an osmolality of ~1,800 mOsm/kg. Beyond these families, multiple species were extremely sensitive to osmolality, including the mucin degrader *Akkermansia muciniphila* ATCC BAA-835, most Prevotellaceae species tested, and Erysipelotrichaceae member *Holdemanella biformis* VPI C17-5 ATCC 27806. While many bacterial strains showed decreased growth rates under high osmolality, they still reached maximum yields similar to those achieved in normal osmolality, indicating the same ability to leverage nutrients in these limiting conditions (Fig. S1B).

Having created a resource of growth abilities for a broad set of gut bacteria, we sought to discover the mechanisms that underlie the tolerance of some bacteria to different osmotic conditions by finding genes or functions that are consistently over-represented in tolerant bacteria. As the majority of genomes (81/92) in our strain library have been fully sequenced and assembled, we performed comparative genomics analyses by combining genomic annotations conducted using tools created by PATRIC with our quantified growth phenotypes (34).

Next, we employed an ML strategy to determine whether potential annotations employed by PATRIC were indicative of a strain’s ability to grow in varying environmental osmolality and pH conditions. Unlike most ML applications, where the goal is to train a model based on input features and then evaluate and optimize its accuracy and generalization, our goal was restricted to the task of feature selection from the large set of available PATRIC annotations. Therefore, we constructed a novel featurization of the PATRIC annotations and used a simple ML model called a decision stump to fit many predictive models to the data. For each candidate PATRIC feature, we measured modeling error on both a training set and held-out test set. We then used the modeling error to rank all the PATRIC features according to their ability to predict the phenotype. This ML analysis efficiently identified PATRIC annotations (ML model features) that correlated with an increased maximum OD of bacterial growth in the different growth conditions (Materials and Methods; Table S2).

We found that the presence of several subsystems correlated with a higher maximum OD at high osmolality (Table S2). A challenge with this type of analysis is that many functional annotations may be correlated and present in the same strains (Fig. S2), but not necessarily directly implicated in response to perturbations. Thus, we identified features that ranked high in distinguishing growth phenotypes and were mechanistically plausible. Importantly, the held-out data set performed comparably to the training data set, indicating the identified features generalized across this sampling of strains (Fig. S3A). To further increase our ability to detect relevant features, we re-analyzed a previously published and annotated metagenomic data set of *in vivo* osmotic perturbation in a humanized mouse model (14). Briefly, in this data set, mice were exposed to the osmotic laxative PEG, which increased the mean intestinal osmolality from 533 to 810 mOsm/kg. The functional pathways present in this community were then quantified for mice prior to, during, and after osmotic perturbation. We identified features that were both over-represented in high osmolality *in vivo* and detected in our ML analysis.

Of particular interest were the subclasses for glutathione biosynthesis/gamma-glutamyl cycle, choline uptake and conversion to betaine, and osmotic stress (Fig. 2B through D). Notably, we also identified cold shock proteins as a distinguishing feature for growth in different osmotic conditions (Fig. S3B and C). Characterized bacterial taxa that possessed annotated genes within these subsystems or roles demonstrated a higher maximum OD on average with increasing osmolality and were also significantly over-represented *in vivo* in high-osmolality conditions. Confirming the importance of stress gene annotations, we identified the osmotic stress gene category as the top predictive feature in this analysis, with a minimum of 10 features in this category being predictive of growth in high-osmolality conditions (Fig. 2D; Table S2). These analyses reveal candidates for future transcriptomic/proteomic studies that will be necessary to define the mechanisms by which these features may support osmotolerance.

### Bacterial families demonstrate a wide range of phenotypic variation in growth and yield in response to acidic/alkaline stress

We assessed the growth of the strain library for a pH range of 4–7.4 and revealed a wide range of tolerances to acidic conditions (Fig. 2A). Because physiological pH within the gut varies with intestinal site, diet, and disease, we chose a range of pH conditions to encompass physiologically relevant perturbations a bacterial species may face (4, 22, 23, 25). Most strains, except for Lactobacillaceae members, were unable to grow at pH 4; even at pH 5.5, these strains displayed serious defects in growth rate and maximum yield. Bacteria within the Lactobacillaceae family displayed the highest tolerance to low pH, as expected for lactic acid bacteria. However, even within this family, genera differed in their tolerance, with several members of *Lactobacillus* and *Ligilactobacillus* displaying sharp decreases in growth rate and maximum OD at pH 4 and inhibition of growth at pH 7.4 compared with physiological pH (6.4–7). Interestingly, isolates from commercial probiotic sources showed high sensitivity to pH changes. Furthermore, at pH 5.5, many species, such as Erysipelotrichaceae members, displayed a decreased growth rate and yield. An exception were members of the Desulfovibrionaceae*,* Fusobacteriaceae*,* Veillonellaceae, and Bifidobacteriaceae families, which contain members that have been described in acidic environments (e.g., acidic mine tailings, dental caries, vagina, and fermented foods, respectively) (35 - 39). Reports have also shown that *Bacteroides* species, which belong to the family Bacteroidaceae, are sensitive to low pH (40); however, we observed a wide range of sensitivities in growth at pH 5.5 in this genus, suggesting that different species and strains may be better adapted to acidic conditions. Some families displayed severe deficits in growth rate and yield at pH 5.5, including members of the Enterobacteriaceae and Streptococcaceae families. An acid-tolerance response has been described for members of the Enterobacteriaceae family (e.g., *Escherichia coli* and *Salmonella*) (41); however, this response may require priming in mildly acidic conditions prior to exposure to more acidic conditions. In our experiments, we subcultured bacteria at neutral pH immediately prior to growth under experimental conditions to simulate the transfer of a healthy microbiota into a diseased environment, which could potentially mask acid-stress adaptations in these families. Interestingly, several bacteria displayed narrow pH tolerances and were inhibited by mildly alkaline conditions, including Lactobacillaceae members. This sensitivity to alkaline conditions has been documented for Lactobacillaceae (19). Interestingly, similar to our observations in high-osmolality conditions, relative deficits in growth rate did not always translate into deficits in final yield (Fig. 2A), suggesting a path for survival of species in communities experiencing physical perturbation in the gut, if they are able to withstand washout.

After identifying growth patterns across our strain library, we once again performed ML analysis on PATRIC features to identify subsystems correlated to bacterial strain growth in acidic/alkaline stress. Unlike our osmolality analysis, the identified PATRIC features were sensitive, in that the model’s fit of the held-out data showed poorer generalization (Table S2; Fig. S4). Furthermore, PATRIC features ranked as highly predictive strongly correlated with specific bacterial families such as Lactobacillaceae, and therefore had less broadly predictive power across the identified strains.

### Taxon-specific responses to pH and osmolality are predictive of behavior in naturally derived complex communities

**Fig 3:**
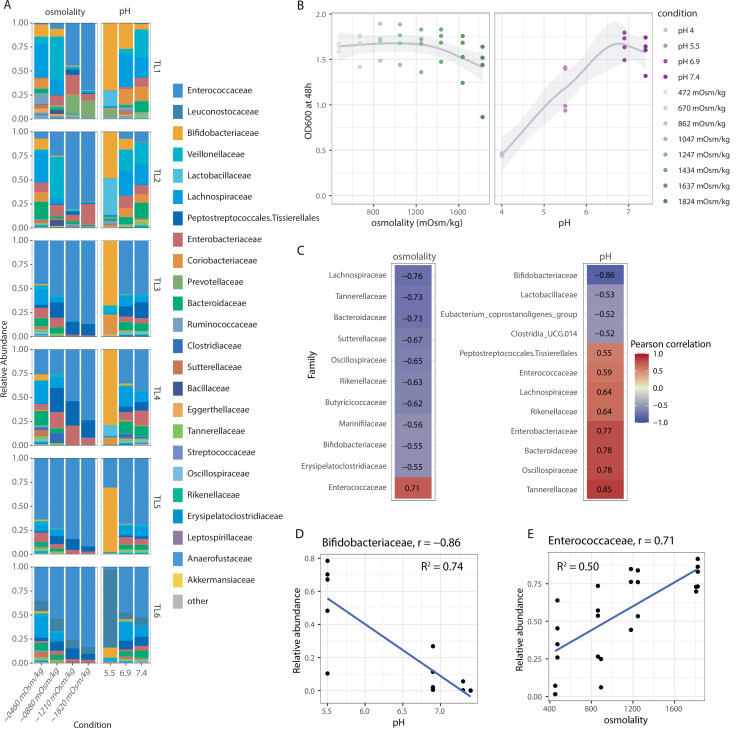
The relative abundance of bacterial families correlates with osmolality and pH across multiple complex microbiota communities *in vitro*. (**A**) Bar plot of relative abundance of bacterial taxa isolated from *in vitro* cultures of human fecal samples (*n* =6) subjected to ranges of pH and osmolalities identical to *in vitro* single-strain cultures. The relative abundance and composition of bacterial families were determined via 16S rRNA sequencing. (**B**) OD_600_ at 48 h, measured to determine the effect of osmolality and pH on community growth. (**C**) Pearson’s correlations of bacterial families in complex communities of human fecal samples characterized *in vitro*, with negative correlations between relative abundance and osmolality (left) and pH (right) highlighted in blue and positive correlations in red. (**D**) Plot of relative abundance as a function of increasing pH for Bifidobacteriaceae, demonstrating a negative correlation between pH and relative abundance (*r*=−0.86). (**E**) Plot of relative abundance and increasing osmolality for Enterococcaceae, demonstrating a positive correlation between osmolality and Enterococcaceae abundance (*r* =0.71).

In the intestine, the microbial communities comprising the microbiota are affected by the physical environment as well as other microbial species that compete for resources and may produce inhibitory molecules (42). To determine whether the behaviors in single-strain pure cultures are generalizable to growth phenotypes in communities, we examined the growth of complex gut microbiota in *in vitro* cultures subjected to defined pH and osmolality environments. We cultured feces from six healthy human donors for 48 h in Mega Medium under the same medium conditions used to assess the single-strain growth of the individual strains. Selection of fecal microbiota from multiple unrelated donors enabled us to study different taxa that are naturally coexisting and adapted to their specific complex community, allowing us to identify generalizable behaviors independent of specific metabolic interactions. Varying pH and osmolality resulted in a wide range of community compositions (Fig. 3A). We found that community OD at 48 h (Fig. 3B) and DNA concentration (Fig. S5) were generally resilient to osmolality except at the highest osmolality tested, but showed a much greater pH dependence. This mirrored the behavior of the most osmotolerant and acid-tolerant species in single-strain growth (Fig. 2), suggesting that these strains drove the observed behavior of the communities. We compared abundance changes in different conditions at the family level, avoiding potential strain-specific behaviors. We observed numerous and distinct positive and negative correlations between pH and osmolality and the relative abundance of specific families (Fig. 3C). A negative correlation with pH indicates that a family is more tolerant of low pH, while a negative correlation with osmolality indicates that a family is less tolerant of high osmolality. These correlations of human taxa for varying pH mirrored our observations in the single-strain growth of individual species, suggesting that, for specific taxa, the environmental pH is a stronger driver of bacterial abundance than the specific microbiota composition. For example, the relative abundance of Bifidobacteriaceae exhibited a highly negative correlation (*r *= −0.86) with increasing pH (Fig. 3D), matching the widespread low pH tolerance of Bifidobacteriaceae species relative to other families (Fig. 2A). In contrast, the Enterobacteriaceae, Tannerellaceae, Oscillospiraceae, and Bacteroidaceae families correlated positively with increasing pH (suggesting acid sensitivity), which is consistent with our single-strain growth data. Responses of bacterial families to different osmolalities in fecal fermentations also displayed similarities to single-strain responses. For example, the relative abundance of Enterococcaceae exhibited a highly positive correlation with osmolality (*r*= 0.71) (Fig. 3E), in line with the tolerance of Enterococcaceae species to high osmolality *in vitro* (Fig. 2A) and *in vivo* (14). Conversely, members of the Lachnospiraceae and Bacteroidaceae families displayed a strong negative correlation with osmolality; the heterogeneity of single-strain responses in these families (Fig. 2A) suggests that the populations within the surveyed communities may be skewed toward relatively osmolality-sensitive members.

For many strains, the single-strain behavior was predictive of their response in a community setting; however, we observed a weak correlation with environmental pH or osmolality for some bacterial families whose members in our strain library displayed consistent responses *in vitro*. For example, some correlations for bacterial species that demonstrated acid tolerance in *in vitro* single-strain growth (e.g., Lactobacillaceae) were not as strong as expected. In some instances, we observed heterogeneity in responses across donors, likely stemming from sparsity of taxa in individual donor samples. For example, Bifidobacteriaceae were the dominant family at low pH in all samples except for one [Tropini Lab 6 (TL6)], in which the lactic acid bacterial family Leuconostocaceae dominated; this family was absent or present at less than 1% in all samples except for TL6. In the case of the Lactobacillaceae, in communities in which it was detected, the pH 5.5 condition supported the highest relative abundance of this taxon; however, Lactobacillaceae were entirely undetectable in three donors, and even in the other donors it was undetectable in conditions other than pH 5.5 (Fig. 3A). This sparsity both across donors and within conditions impacted the calculated correlations. Additionally, other factors, such as nutritional or resource competition, may contribute to the relative abundance of bacterial species in the intestine (Fig. 3C). Finally, the Enterobacteriaceae family displayed increased abundance in increasing osmolality in only some fecal communities (TL1, TL2, and TL4), which may be due to genus- or species/strain-level variation or, more likely, out-competition by other osmotically tolerant bacteria present in non-responsive fecal communities. Because we used 16S ribosomal RNA (rRNA) amplicon sequencing and measured relative abundances, the survival and proliferation of other osmotically tolerant bacteria could mask survival or increases in absolute Enterobacteriaceae abundance.

### Single-strain behavior correlates with responses to environmental pH and osmolality in the gut

**Fig 4:**
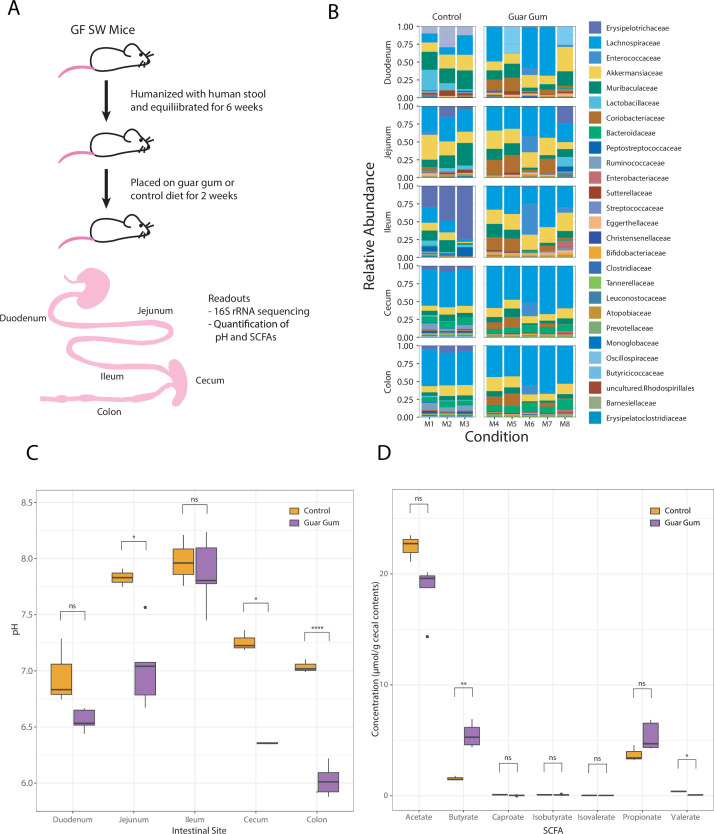
Humanized mice on a guar gum diet demonstrate a significant drop in cecal pH and shifts in bacterial family composition. (**A**) Experimental schematic of germ-free (GF) SW mice, detailing the timeline of humanization and the switch to a guar gum diet (*n* = 5) versus a control diet (*n* = 3). The bottom figure depicts segments of the gastrointestinal tract collected for pH measurements, 16S sequencing, and SCFA measurements. (**B**) Relative bacterial abundance of humanized mice on the guar gum or control diet, highlighting the gut microbial composition at the family taxonomic level. (**C**) Quantification of pH along the gastrointestinal tract of humanized mice on the guar gum or control diet. (**D**) SCFA concentrations in cecal contents from humanized mice on the guar gum or control diet.

Having shown the *in vitro* generalizability of pH and osmolality resilience in a complex microbiota for several key taxa, we explored whether these phenotypes would also be consistent *in vivo*, where, beyond microbiota interactions, the interplay with host dynamics plays a significant role in determining bacterial abundance. As other studies have investigated the response to osmolality *in vivo* (14, 43), we sought to investigate how microbiota members are affected by changes in pH *in vivo*. We reasoned that changing diet would impact gut pH differentially in the various intestinal compartments (44, 45). Specifically, microbial fermentation of carbohydrates in the cecum and colon produces SCFAs, lowering the pH of these intestinal compartments. As with any perturbation *in vivo*, changing diet will have multiple orthogonal effects (i.e., in this case, combining differences in nutrient availability for both the host and the microbiota as well as pH); however, using our *in vitro* analysis, we reasoned this model might enable us to identify patterns that are consistent with pH tolerance. Although most monosaccharides and disaccharides are hydrolyzed and absorbed in the small intestine, many dietary oligosaccharides and polysaccharides cannot be hydrolyzed by host enzymes and pass undigested into the large intestine, where they are readily fermented by bacteria. One such carbohydrate is guar gum (46), a galactomannan polysaccharide comprising a linear backbone of β-1,4-linked mannose residues with randomly attached β-1,6-linked galactose residues, a structure that cannot be digested by the mammalian host (47). We hypothesized that mice on this diet would undergo increased fermentation and acidification in their large intestines relative to mice on a standard diet. We gavaged germ-free Swiss Webster (SW) mice with feces from a healthy human donor, colonizing their intestines with a representative community of human intestinal bacteria (Fig. 4A). After equilibration for 6 weeks on a standard rodent diet, the mice were divided into two groups: one group was placed on a diet with 30% guar gum and allowed to adjust to their diet for an additional 2 weeks, whereas the second group continued on a standard rodent diet (Fig. 4A). We then sacrificed the mice and performed 16S rRNA sequencing (Fig. 4B) and pH measurements (Fig. 4C) on different intestinal segments. Measurements of intestinal content revealed a significant decrease in pH in the jejunum, cecum, and colon of mice receiving the guar gum diet (Fig. 4C), suggesting that increased fermentation occurred. Unlike the pH at the other sites, the duodenal and ileal pH values were unaffected by the diet change. We then investigated whether SCFA production in the cecum was altered by the diet. Indeed, we found that butyrate levels increased threefold in mice fed the guar gum diet while other SCFAs were not significantly affected except valerate, which was mildly decreased (Fig. 4D).

Next, we analyzed the relative abundance of bacteria in the different intestinal segments and diet conditions by performing 16S rRNA sequencing of communities isolated from those regions. In mice on the guar gum diet, the average cecal and colonic pH values were 6.36 and 6.02, respectively, compared with 7.26 and 7.04 for the standard-diet-fed mice (Fig. 4C). Given the distinct pH tolerance profiles of different bacterial families (Fig. 2A), we hypothesized that this acidification would change the composition of the colonic microbiota. We observed an increase in the butyrate producer *Blautia* (Lachnospiraceae family) and a loss of the Erysipelotrichaceae family (Fig. 4; Fig. S6). Our single-strain analyses revealed that *Blautia* was able to grow at pH 5.5, consistent with its ability to thrive in these conditions *in vivo* (Fig. 2A and 4B). Conversely, our *in vitro* data suggest that many *Bacteroides* members are sensitive to low pH (Fig. 2A). Interestingly, we observed *in vivo* expansion of *Bacteroides* within the cecum and colon in experimental mice despite *Bacteroides* members displaying a sensitivity to *in vitro* conditions of pH 5.5 and below (Fig. 2A and 4B). This observation suggests that the expansion of relatively more acid-tolerant *Bacteroides* isolates within the same niche may occur upon changes in the physical environment.

## DISCUSSION

In this study, we sought to characterize the pH and osmolality tolerance of a wide range of publicly available and sequenced gut bacteria and probiotic strains. We characterized the growth of 92 bacterial representatives of the gut across 28 families under multiple pH and osmolality conditions *in vitro* (Fig. 1A). Most of these bacterial species were human isolates, selected to maximize their relevance to the human intestine; however, by measuring the response to pH and osmolality in both mouse and human isolates from the same families, we provided a more extensive coverage of the diversity found within gut bacteria across multiple hosts. We developed high-throughput growth assays to test bacterial growth in pH and osmolality conditions representative of those found within the gut in health and disease. Our measurements demonstrate a wide range of tolerance to perturbation across bacterial families and family specific responses to changes in pH and osmolality. Within families, individual members across hosts demonstrated a varied response to *in vitro* conditions. Using comparative genomics, we uncovered the abundance and prevalence of genes in characterized microbial taxa responding to stress. In many cases, the abilities to tolerate acidic stress and high osmolality were congruent with the abundance and presence of identified stress response genes. One limitation of this study arises from differences in the depth of gene annotations/knowledge of the more deeply studied families, such as Enterobacteriaceae and Bacteroidaceae, and more newly discovered and relatively culture-intractable families (Fig. 2A). However, our analysis provides a framework for identifying strains that may possess novel stress responses for cases in which an isolate shows growth in limiting pH or osmolality conditions despite not possessing annotated stress tolerance genes.

We observed widespread pH and osmolality tolerance in representatives of Lactobacillaceae (including isolates from commercially available probiotic sources) and Enterococcaceae, respectively. Conversely, Bacteroidaceae and Bifidobacteriaceae displayed heterogeneity in their responses to osmolality and pH. Despite a broad diversity of growth phenotypes, we did not observe genomic features that could explain the phenotypic variation. This may be due to a difference in unannotated genes, or in the expression of annotated genes, and suggests that transcriptomic analyses or single-gene knockout libraries may be required to untangle mechanisms underlying the differential tolerance of these bacteria.

Interestingly, many strains that displayed deficits in maximum growth rate at lower pH or higher osmolality still produced similar maximum yields (Fig. S1B). Although bacteria in the intestine must grow at a sufficient rate to prevent washout, maintaining a maximum efficiency of biomass yield may also be an effective strategy for survival; thus, efforts to connect single-strain tolerance to behavior/yield in complex communities must incorporate both metrics. It is also important to note that while some of these strains displayed relative deficits in growth rate compared with their maximal growth rate in ideal conditions, a strain merely needs to survive or efficiently produce biomass relative to other bacteria to be propagated in the gut. Moreover, some bacterial taxa spatially inhabit specific niches within the gut, which limits the competition for resources against other bacteria preventing washout.

Our ML analysis highlighted how the presence of specific genes is strongly predictive of growth at different osmolalities (Fig. 2B through D). Many of these genes are involved in stress tolerance (48, 49). For osmotic stress, the subsystem of glutathione biosynthesis/gamma-glutamyl cycle has previously been shown to provide osmo-adaptation (48). Tellingly, mutants lacking genes within this subsystem (e.g., *gshA* and *gor*) in *E. coli* show deficient growth in elevated osmolarity (48). Furthermore, we identified other genes not traditionally viewed as osmotic-stress response genes (Table S2). For example, we found that dihydrolipoamide dehydrogenase is predictive of osmotic tolerance. This protein functions to oxidize dihydrolipoamide in a ping-pong mechanism as an oxidoreductase (50). Interestingly, the identified dihydrolipoamide dehydrogenase of the pyruvate dehydrogenase complex has been implicated in the increased osmo-tolerance of *Staphylococcus aureus* (51, 52).

The identification of genes and pathways involved in pH tolerance was less obvious than that for osmolality, indicating that family specific tolerance mechanisms may be at play, masking potential generalizable features. This finding indicates that community-based techniques, such as metagenomics, may not shed light on the importance of specific genes involved in pH tolerance; identified features that may be unrelated to pH may be over-represented in acid-tolerant bacteria and appear significant. Therefore, we predict that more traditional genetic screens and transcriptomics assays will be needed to discover genes involved in pH tolerance in poorly annotated bacteria. For cases in which there is phenotypic variation within a family, comparative genomic techniques may be valuable, but for cases in which there is complete penetration of a phenotype within a taxon, elucidation of such features will prove more challenging. Importantly, we also found that the most acid- and osmolality-tolerant bacteria generally did not overlap (Fig. 2), suggesting that there are distinct mechanisms for acid and osmolality tolerance. This was also confirmed in our ML analysis, in which the set of features that predicted osmolality tolerance did not rank highly in pH tolerance (Fig. S4C).

We then explored whether single-taxon phenotypes are generalizable to a complex microbiota grown in complex media (Fig. 3). Using six distinct human fecal microbiota samples, we tested growth across pH and osmolality conditions and found that growth patterns observed in isolated bacteria were consistent with relative abundance in complex microbiota communities. Bifidobacteriaceae and Bacteroidaceae were negatively and positively correlated with pH, respectively, in concordance with their acid tolerance and sensitivity in single-strain growth (Fig. 2A). Similarly, families such as Enterococcaceae exhibited positive correlations with osmolality, mirroring their tolerance to osmolality. Enterobacteriaceae also displayed osmotic tolerance in pure cultures and in three donor samples (Fig. 3; TL1, TL2, and TL4); this family flourished in intermediate and high osmolalities as well. The lack of significant correlation across donors may be due to several reasons, including genetic or phenotypic variation at the species level and out-competition by highly osmo-tolerant bacteria such as Enterococcaceae. Although single-strain response data may highlight tolerance and potential mechanisms for survival, the *relative* tolerance and resilience of competing bacteria may ultimately be the determinant for success. Sparsity of taxa across donors may also underlie the lack of obvious correlations in some taxa; for example, we observed a bloom in the lactic acid bacteria family Leuconostocaceae in one donor sample (Fig. 3; TL6) at pH 5.5; this species, which is found in fermented food products, was absent in three donors and present at approximately 0.1% or less in TL3 and TL5, where it appeared that Bifidobacteriaceae dominated in low pH conditions. Similar sparsity between donors and within conditions underlies the weak correlation between Lactobacillaceae and pH despite its higher abundance at low pH. This result underscores the importance of quantifying tolerance across multiple bacterial families to capture the potential diverse responses in heterogenous human populations.

Finally, we investigated whether our *in vitro* observations were representative of an *in vivo* microbiota responding to changes in gut pH (Fig. 4). We selected an animal model with a dietary intervention that yielded a decrease in pH in multiple intestinal segments and a significant increase in butyrate, a SCFA found in cecal contents. These changes corresponded to a loss of Erysipelotrichaceae, a family that displayed extreme sensitivity to pH in single-strain growth with member *H. biformis* (Fig. 2A). Additionally, our *in vitro* strain characterizations confirmed the *in vivo* phenotypes, we previously observed in an animal model of mild osmotic diarrhea induced by PEG laxatives (14). In our *in vitro* strain library, the families Enterobacteriaceae and Enterococcaceae displayed high osmotic tolerance; these families experienced significant expansions during PEG treatment in humanized mice (14). Similarly, the family Verrucomicrobiaceae (which contains the species *A. muciniphila*) was extremely sensitive to osmolality in our *in vitro* characterization; this species decreased 25-fold during osmotic perturbation *in vivo* (14).

Taken together, these results highlight the importance of the physical parameters of pH and osmolality and their role in the survivability of bacterial taxa found within the gut and, therefore, overall gut community composition. By quantifying the pH and osmolality tolerance across a wide range of representative intestinal bacterial families, we found that *in vitro* tolerance to physical parameters in single-strain growth can predict the effect of changes on complex communities in an *in vivo* physical environment. The tolerances we investigated were also consistent in *in vivo* animal models for multiple taxa. Thus, quantifying taxon-specific responses of the gut microbiota to environmental perturbations provides key information regarding the dynamics of community changes during health and disease.

Beyond the consistency between individual species growth and community relative abundance patterns in different environmental conditions, a valuable implication of our studies is the importance of broadly characterizing the physical environment in microbiota studies. To better understand how to remediate diseases associated with both dysbiosis and environmental perturbation of the gut such as IBD, it is crucial to establish the physical parameter ranges in healthy and perturbed environments, including the microenvironments along the intestine that are relevant to disease states. Once these environmental parameters have been quantified, efforts can be made to predict how the existing pH and osmolality may affect the survival of prospective communities in the gut. These measurements are also important in microbiota therapies, where the tolerance of probiotic strains to osmotic and acidic perturbations must be identified to determine survivability and potential function as a therapeutic within a perturbed gut environment. Potential probiotic strains must be able to propagate and compete against resident microbes in an environment to provide therapeutic effects. Our work has demonstrated that probiotics within a family may differ in their tolerance to physical parameters; we observed strong heterogeneity among Lactobacillaceae (Fig. 2A), which contains many currently marketed probiotic strains. For a diseased environment in which physical parameters are altered or misregulated, our results suggest these probiotics may not fare equally well. Thus, the selection of a particular strain must consider physical measurements from the disease state of interest as well as the tolerance of potential therapeutic probiotics.

Overall, these results indicate that the physical environment is a key predictor of bacterial abundance over a broad range of conditions and across multiple communities. This predictability across physiological ranges highlights the importance of monitoring the physical environment in microbiota studies as a key driver of bacterial availability and the utility of determining the diverse individual responses of bacteria in single-strain cultures.

## MATERIALS AND METHODS

### Phylogenetic tree construction

We acquired 16S sequences for most bacterial species from the SILVA database (https://www.arb-silva.de/search/) and the National Center for Biotechnology Information (NCBI) (Project ID: PRJNA474907). Sequences downloaded from SILVA were at least 1,500 bp in sequence length. The downloaded FASTA files were compiled into a single file and imported to MEGA 11.0.10: Molecular Evolutionary Genetics Analysis version 11 for alignment using the MUSCLE algorithm and for construction of a phylogenetic tree using the “Construct/Test Neighbor-Joining Tree” option (53, 54). We then uploaded the Newick file generated by MEGA 11.0.10 to iTOL v6 (https://itol.embl.de/) for modification and coloring (53).

### Bacterial culture

The bacterial strains and corresponding metadata (i.e., taxonomy) used in this study are reported in Table S1. All bacterial strains were grown and inoculated in a vinyl anaerobic chamber (Coy Laboratories, Grass Lake, MI, USA) maintained with an atmosphere of 5% CO_2_, 5% H_2_, and 90% N_2_ (Linde Canada, Delta, BC, Canada). All strains were incubated at 37°C for growth, and all glycerol stocks were stored at −80°C.

### Bacterial media

We prepared Mega Medium using the protocol provided in the Supplementary Methods, with minimal modifications from a previous publication (31). Each batch of liquid and solid media was autoclaved and pre-reduced in an anaerobic chamber at least 24 h before use. To characterize the pH and osmolality tolerance of strains, we aseptically loaded liquid Mega Medium into two sterile 96-deep-well plates, with media adjusted to eight different conditions. Medium conditions consisted of Mega Medium adjusted to pH 4, 5.5, 6.9, or 8 (osmolality normalized to ~600 mOsm/kg, the osmolality of the pH 4 condition) or to osmolality conditions of ~440, ~890, ~1,176, or ~1,800 mOsm/kg. We adjusted medium osmolality conditions using sodium chloride. Lowest osmolality condition of adjusted media was dependent on the basal osmolality of media used to characterize strains ranging from ~234 to ~440 mOsm/kg. We adjusted the medium pH using 10 N HCl and NaOH (33 wt% solution in water). For pH measurements, we calibrated a micro pH probe (Orion PerpHecT ROSS Combination pH Micro Electrode, Catalog number: 8220BNWP) for media adjustment. For osmolality measurements, we injected 20 µL of media into an Advanced Instruments Osmo1 Single-Sample Micro-Osmometer using an Ease-Eject 20-µL Sampler and clean sampler tips. We filter-sterilized the media using 150 mL 0.22 µm vacuum filtration tops (VWR: 10040-444) after adjustment. Due to the relatively high base osmolality of the pH media, some strains could not grow in any pH conditions and were therefore grayed out in the heatmap (Fig. 2A). We loaded one plate with media containing 1 µg/mL BCECF (Thermo Fisher Scientific, Waltham, MA, USA) to measure the environmental pH during growth. BCECF is a fluorescent pH sensor that detects extracellular changes in medium pH by establishing a pH-dependent ratio of emission intensity at excitation wavelengths of 440 and 490 nm. We loaded another plate without BCECF to accurately determine the BCECF signal during pH calculations. Both plates were incubated in an anaerobic chamber for 24 h to pre-reduce and equilibrate the media for anaerobic growth. For some strains that were unable to grow in Mega Medium, we used solid and liquid media of peptone yeast glucose (PYG) medium, brain heart infusion-supplemented (BHIS) medium+mucin, and Mega Medium supplemented with either lactate or a combination of sodium citrate and MgSO_4_, as noted in Table S1.

### Bacterial growth

Bacterial isolates were streaked onto solid media to isolate individual colonies from glycerol stocks and were grown at 37°C; single colonies were picked after 24–48 h and cultured in “overnight” pre-reduced liquid media at 37°C for 16–36 h. We primarily used Mega Medium for strain characterization, with a few exceptions (Table S1). Overnight cultures were diluted 10-fold in pre-reduced liquid media and incubated at 37°C for 2 h. We then diluted the cultures fivefold into pre-reduced liquid media in a 96-well plate in preparation for high-throughput strain loading. Subsequently, we added 5 µL of cultures to 75 µL of each medium condition in a 384-well plate, resulting in a 16-fold dilution with an 80-fold dilution in total. Each 384-well plate consisted of eight conditions varying in pH and osmolality, with each strain grown in quadruplicate in each condition. Three of these replicates contained BCECF, and one replicate was BCECF-free, which enabled real-time environmental pH measurements coupled with OD measurements. Cultures grew at 37°C, and we measured the absorbance at 600 nm and BCECF fluorescence every 13 min using a Synergy H1 plate reader (BioTek Instruments, Winooski, VT, USA) for 48–96 h of growth. We measured BCECF fluorescence using excitation wavelengths of 440 and 490 nm detected at 535 nm for every time point on the growth curve. To quantify the medium pH, we determined the ratio of emission intensity between excitation at 490 nm versus 440 nm and calibrated this ratio to a calibration curve of pH values measured before each experiment, according to Invitrogen’s protocol. We conducted our analysis using a custom-made MATLAB program (https://github.com/Tropini-lab/Strain_library_paper).

### Growth analysis

Growth curves were run through a custom MATLAB script (https://github.com/Tropini-lab/Strain_library_paper). Briefly, the program identifies replicates based on assigned metadata (strain, pH/osmolality, etc.) and automatically selects the three most similar replicates for each condition for averaging and plotting. The maximum growth rate for each OD curve is determined after a least-squares fit is performed for the OD curve to the Gompertz equation (54).

### Growth data standards

We selected appropriate growth data for strains grown in each condition by comparing growth data against control conditions. Controls consisted of sterile Mega Medium (or BHIS+mucin, PYG, or Mega Medium supplemented with lactate or MgSO_4_+sodium citrate); strains that increased in OD at the same time as or after control wells were discarded and re-run in subsequent experiments. For bacterial strains that were selected and considered clean, we performed outlier detection on the quadruplicate OD measurements and selected the best three out of four technical replicates in each condition for downstream analysis.

### Isolation of commercial probiotics for characterization

We dissolved probiotics purchased from local pharmacies in sterile 1× phosphate-buffered saline (PBS; Fisher Bioreagents: BP3991) in the anaerobic chamber. The dissolved slurry was streaked onto agar plates and incubated anaerobically at 37°C for 24 h. Both PBS and medium were pre-reduced in an anaerobic chamber for at least 24 h before use. We isolated Lactobacillaceae using Mega Medium and Bifidobacteriaceae using *Bifidobacterium* selective iodoacetate mupirocin medium according to a previously published method (Table S1) (55).

### Stock preparation

We obtained bacterial isolates from multiple culture collections, including BEI, ATCC, and DSMZ. Source cultures were streaked onto Mega Medium agar or appropriate media as noted in Table S1, and single colonies were picked and frozen for storage using a 1:1 mixture of culture and a 50% glycerol solution. The solid- and liquid-rich media used for stock production are listed in Table S1. We confirmed the purity of final cultures via Sanger sequencing of the 16S rRNA gene using 8F and 1391R primers (8F: 5′-AGAGTTTGATCCTGGCTCAG-3′, 1391R: 5′-GACGGGCGGTGWGTRCA-3′).

### PATRIC annotations

Genomes of publicly available species were downloaded from NCBI and submitted to PATRIC (https://www.patricbrc.org/) for annotation (56). The NCBI taxonomy ID and domain (i.e., bacteria) are required for submission. The abundance of annotated genomic features was compared across species at subsystem levels using the Shiny library in RStudio.

### *In vitro* growth and PATRIC subsystem analysis

Analyses and graphing were performed using R v4.1.2 and RStudio v1.4.1717. We conducted heatmap analyses of RAST (rapid annotation using subsystem technology) subsystems and growth data through an in-house-developed R library named “strains_heatmaps” (available for download in the following GitHub repository: https://github.com/Tropini-lab/Strain_library_paper) and the ComplexHeatmaps package v2.10.0 (57, 58). Briefly, our R library assembles all tables downloaded from RAST into a data frame that compares the number of features present in the different strains. Then, the R library filters and collapses the data frame based on broad annotation categories (in our case, subsystems involved in acid/osmolality tolerance) and transforms the data frame into a format compatible for use with the ComplexHeatmaps library. The ComplexHeatmaps library is then implemented to make various heatmaps, with coloring based on feature counts for each subsystem for each strain and the growth data joined as additional heatmaps or heatmap annotations. For more detailed explanations, please refer to the scripts and tutorials in the GitHub repositories (https://github.com/Tropini-lab/Strain_library_paper).

### Machine learning

The goal of our ML model was to determine that PATRIC annotations could predict a strain’s ability to grow in varying pH and osmolality conditions.

#### Model input feature preparation

Using Python (version 3.10.5), we constructed a tabular Pandas (version 1.4.3) DataFrame of features for each strain’s genome starting from the PATRIC subsystem annotation output (59). PATRIC maps the strain genome name (*genome_name* in Table S3) to many PATRIC IDs, each of which is annotated with a *Superclass*, *Class*, *Subclass*, *Subsystem* name, and *Role ID*. Because this is a one-to-many mapping of *genome_name* to PATRIC ID, each *genome_name* appears on multiple rows, with various numbers of PATRIC IDs for each *genome_name*.

To efficiently fit the ML model, we used a fixed-length list of numbers representing each *genome_name*, that is, a feature vector. To featurize the 81 sequenced *genome_names* using the PATRIC annotations, we counted the number of times a particular value occurs in the PATRIC *Superclass*, *Class*, *Subclass*, *Subsystem* name, and *Role ID* columns for a given *genome_name*.

For example, if a particular *genome_name* maps to exactly seven PATRIC IDs for which the *PATRIC Subsystem* column’s value is “DNA repair, bacterial,” we create a feature column named “Subsystem Name = DNA repair, bacterial” whose feature value on the row for that *genome_name* is 7.

We further annotated the featurized DataFrame with additional feature columns for each *genome_name* with binary indicator variables for its location within the phylogenetic tree. For example, there was a column named “Family = Bacteroidaceae” whose value was 1 for every *genome_name* in the Bacteroidaceae family and 0 for other genome names. We added these indicator variables for all observed values in the *Phylum*, *Class*, *Order*, *Family*, *Genus*, and *Species* columns.

The feature DataFrame is relatively sparse and is thus filled with many cells containing counts of 0, as many *genome_names* did not associate with values for the PATRIC columns whose values we counted, but we did not take advantage of this sparsity.

#### Model output values

We joined the growth data outputs (maximum OD across pH and osmolality conditions; Table S4) for each strain’s genome with the information obtained based on the *genome_name*.

We constructed separate models for predicting pH and osmolality responses. Within those models, we jointly modeled all observations relevant to each perturbation. For example, we constructed a single model that jointly predicts the normalized maximum OD observations across all pH conditions based on the above-described features representing the *genome_name*. Thus, our models for predicting pH response have four real-valued outputs for the observations at pH=4, 5.4, 6.7, and 7.3. Likewise, the osmolality prediction models have four outputs, for the lowest osmolality, 890, 1,180, and 1,800 mOsm/kg.

The osmolality measurements for one of the *genome_names* failed at two osmolality values; hence, we removed this row from the DataFrame used to fit the osmolality models, leaving 79 rows. The pH data had only one failed row.

#### Model architecture, loss function, and training procedure

Our featurized Pandas DataFrame was very short and wide (81×11,514), with a single row for each of the 81 different *genome_names* and 11,514 different feature columns. This shape is atypical for ML applications due to the potential for overfitting, but we were interested in feature evaluation rather than precise modeling; thus, we used a one-level decision tree regression model, also known as a decision stump (60). We fit this model using sklearn’s default parameter values for regression trees, which minimizes the total squared error on both sides of the decision stump (60, 61).

#### Decision stump squared-error equation

A decision stump divides a data set into two groups using a single numerical feature and a splitting threshold. Rows in which the feature value is smaller than the threshold go down the left branch of the tree and arrive at the left terminal leaf node, whereas rows for which the feature value is at least as large as the threshold go to the right terminal leaf node.

At each of the left and right leaf nodes, the fitting algorithm computes the mean value for each of the outputs using the rows assigned to that node, which serves as the model’s prediction for all rows with the same classification. The fitting algorithm attempts to select a feature and splitting threshold which minimizes the total squared error of each row’s distance from its associated mean. Pedregosa et al. describe this algorithm as minimizing the “mean squared error, which is equal to variance reduction as feature selection criterion and minimizes the L2 loss using the mean of each terminal node” (61).

#### Single-feature decision stumps

To identify the quality of each candidate feature, we trained a single-feature decision stump on all 11,514 individual features. Because the decision tree fitting code is not required to select between competing features for the root split, the only remaining task is to identify the splitting threshold that minimizes the total squared error across the left and right terminal leaf nodes in predicting the maximum OD across all four responses. Note that this approach places us in the regime of a decision tree regression multi-output problem (61).

#### *K*-fold cross-validation

Even though the single-feature decision stump is a relatively simple model, it is still possible to overfit the data. The large number of features being evaluated increases the chance that this will happen for some features. To mitigate this risk, we performed *K*-fold cross-validation for all the models we fit, using sklearn’s default parameters settings (*K* = 5). *K*-fold cross-validation works by dividing the shuffled data into five partitions. Each time a model is fit, one of the partitions is held out of the training set. We then evaluated the trained model’s squared error on both the training partitions and the held-out test set partition.

Because we trained *K* = 5 different models for each feature, we computed five different estimates for each feature’s squared error on both the train and test sets. To compute an overall estimate of each feature’s quality, we took the mean of those five estimates.

We note that, particularly in modeling the pH data, some features happen to result in low squared error on the test set, despite having relatively high error on the train set (Fig. S4B). To further mitigate this, we computed each feature’s rank among all the features in the train and test set scores, and ultimately ranked the features according to its maximum (worst) rank across the train and test set scores.

Finally, since we trained five different models for each feature, each of the models could have selected a different decision splitting threshold for that feature. For binary partitions of the data used to make box plots and heatmaps, we took the mean value of the decision threshold selected for each of the five models.

### Colony polymerase chain reaction and confirmation of strains used in experiments

Here, 1 mL of overnight cultures grown for 24 h at 37°C was spun down for 5 min at 5,000 × *g* (relative centrifugal force), and the collected pellets were boiled for 5 min. We prepared 1:10 and 1:100 dilutions using sterile DNase-free water to prepare for colony polymerase chain reaction (PCR) [(98°C 2 min) → (98°C 30 s, 57°C 30 s, 72°C 45 s) × 30 cycles → (72°C 10 min) → (4°C ∞)] and performed sequencing using 8F and 1391R primers (8F: AGAGTTTGATCCTGGCTCAG, 1391R: GACGGGCGGTGWGTRCA). Some cultures required DNA extraction prior to PCR, which was performed using the Qiagen DNeasy Blood and Tissue Kit (Catalog number: 69504).

### Human fecal sample collection, fermentation, and DNA extraction

We collected feces from six individuals (TL1, TL2, TL3, TL4, TL5, and TL6), either a day prior to or on the morning of experimentation. We stored all fecal samples in sterile conical tubes at −80°C before processing. Fecal samples (1.5 g) were resuspended in sterile pre-reduced 1 × PBS, and contents were allowed to settle to collect liquid. Liquid Mega Medium was pre-reduced in an anaerobic chamber environment with 5% CO_2_, 5% H_2_, and 90% N_2_ for at least 24 h. For each sample, 1 µL of supernatant was collected and subsequently inoculated into 200 µL of pre-reduced liquid Mega Medium adjusted to a pH of 4, 5.5, 6.9, or 7.6 and an osmolality of 472, 670, 862, 1,047, 1,247, 1,437, 1,637, or 1,824 mOsm/kg on two 96-well plates. We measured the OD at a wavelength of 600 nm (OD_600_) using a BioTek Synergy H1 Plate Reader after anaerobic incubation at 37°C for 48 h. We selected the physical conditions based on generated OD_600_ measurements and extracted DNA from the 96-well plates using the DNeasy PowerSoil Pro Kit (Catalog number: 47016).

### Humanized mice supplemented with a guar gum diet

Germ-free SW mice were gavaged with a human gut microbiota (TL1) at 9 weeks and placed on a standard rodent diet (LabDiet 5k67). Six weeks after colonization, five SW mice (two males and three females) were switched to a guar gum diet (TestDiet 5BSE) for 2 weeks, while three (three males) remained on the standard diet. In Fig. 4B, M1 and M2, and M3 were three male mice and were co-housed and given a standard diet. M4 and M5 were males and co-housed, while M6, M7, and M8 were females that were also separately co-housed. M4–M8 were given a guar gum diet. Two weeks after equilibration on the diet, the mice were sacrificed using carbon dioxide with secondary cervical dislocation. We collected contents from the duodenum, jejunum, ileum, cecum, and colon in the gastrointestinal tract for pH and osmolality measurements. Collected mouse intestinal contents were stored in 1.5-mL microcentrifuge tubes and kept on ice during preparation for pH and osmolality measurements. The same micro pH probe (Orion PerpHecT ROSS Combination pH Micro Electrode, Catalog number: 8220BNWP) and Advanced Instruments Osmo1 Single-Sample Micro-Osmometer were used to measure intestinal contents as described above. In addition, we performed DNA extraction as described above for 16S rRNA sequencing and sent cecal contents for SCFA analysis.

### 16S sequencing library preparation and sequencing

We quantified the extracted DNA using the Quant-iT 1 × dsDNA HS (High-Sensitivity) Assay kit (Catalog number: Q33232). We submitted the DNA samples to either Biofactorial, a high-throughput biology facility located in the Life Sciences Institute at the University of British Columbia, or the Gut4Health Microbiome Core Facility at the British Columbia Children’s Hospital Research Institute and the University of British Columbia for a paired-end sequencing run using a MiSeqv3-600 instrument with dual-indexed V4V5 primers (Biofactorial) or V4 primers (Gut4Health). For samples sequenced at Biofactorial, a dual-indexing, one-step 10 µL PCR reaction was performed on a LabCyte Access Workstation using Quanta repliQa HiFi ToughMix with 0.5 ng input DNA and complete “fusion primers” that include Illumina Nextera adaptors, indices, and specific regions targeting the V4/V5 region of the 16S rRNA genes (62). After quantification of amplicons via a picogreen assay (Quant-iT PicoGreen dsDNA Assay Kit, Thermo Fisher Scientific, Waltham, MA, USA), 2 ng of each product were pooled for subsequent cleanup using the AmpureXP PCR cleanup protocol (Beckman Coulter, Brea, CA, USA). The pooled library was quantified by a picogreen assay and loaded onto an Illumina MiSeq Reagent Kit v3 (600-cycle) according to the manufacturer’s recommendations with 15% PhiX. Samples submitted to Gut4Health were prepared according to a previously published method (63). Briefly, the V4 region of the 16S rRNA gene was amplified with barcode primers containing the index sequences using a KAPA HiFi HotStart Real-time PCR Master Mix (Roche, Grenzach-Wyhlen, Germany). We monitored PCR product amplification and concentration on a Bio-Rad CFT Connect Real-Time PCR system. Amplicon libraries were then purified, normalized, and pooled via a SequalPrep normalization plate (Applied Biosystems, Foster City, CA, USA). We further purified the pooled library with the Agencourt AMPure XP system (Beckman Coulter, Brea, CA, USA) according to the manufacturer’s protocol. Library concentrations were verified by a Qubit dsDNA high-sensitivity assay kit (Invitrogen, Carlsbad, CA, USA) and the KAPA Library Quantification Kit (Roche, Grenzach-Wyhlen, Germany) according to the manufacturer’s instructions. We submitted the purified pooled libraries to the Bioinformatics+Sequencing Consortium at the University of British Columbia, which verifies DNA quality and quantity using a high-sensitivity DNA kit (Agilent) on an Agilent 2100 Bioanalyzer. Sequencing was performed on the Illumina MiSeq v2 platform with 2×250 paired end-read chemistry.

### 16S rRNA data analysis

We imported 16S rRNA reads generated through the MiSeq v3-600 run to QIIME 2 2020.11 for analysis (64). The read quality was assessed by FastQC (https://github.com/s-andrews/FastQC). We processed the sequences using DADA2 (https://github.com/qiime2/q2-dada2). The primers were trimmed, and sequences with a quality score below 30 were truncated. We taxonomically classified the denoised sequences using a SILVA v138-trained classifier (65).

### SCFA analysis of murine cecal samples from the guar gum diet

To prepare SCFA samples for gas chromatography analysis, we extracted SCFAs from 40 to 100 mg of cecal contents. The samples were mixed and homogenized with 800 µL of 25% phosphoric acid. We collected the supernatants via centrifugation at 15,000 × *g* for 10 min at 4°C. We then added 200 µL isocaproic acid and 0.2 mL 25% phosphoric acid as an internal standard. Supernatants were sent to the AFNS Chromatography Facility at the University of Alberta for quantification. We ran the samples on a Varian 430 gas chromatograph with a Stabilwax-DA column (length: 30 m, inner diameter: 0.53 mm, and film thickness: 0.5 µm) and helium carrier gas, using a 250C injector with a split ratio of 5 and a 1 µL injection. We utilized a flame ionization detector with a detector temperature of 250°C. Retention times were compared with known standards.

### Software and algorithms

In this work, we utilized MATLAB 2020a (MathWorks, Natick, MA, USA) to analyze the bacterial growth, as described above. We used BioTek GEN5 software to collect bacterial absorbance and fluorescence. We employed RStudio to compare the abundance of annotated genomic features across species at subsystem levels (Shiny library) in RStudio. We performed analyses and graphing using R v4.1.2 and RStudio v1.4.1717, as described above.

## Data Availability

The data sets generated and/or analysed during the current study are available in the Borealis repository.

## SUPPLEMENTAL MATERIAL

Supplemental material mbio.00753-23-s0001.docx Figures S1 to S6 and captions for Tables S1 to S4.

Table S1 mbio.00753-23-s0002.xlsx Medium components for growth media used in the study.

Table S2 mbio.00753-23-s0003.xlsx PATRIC features identified by ML to distinguish growth in different osmolality and pH conditions.

Table S3 mbio.00753-23-s0004.xlsx PATRIC features used in the ML model.

Table S4 mbio.00753-23-s0005.xlsx Normalized growth rate (GR) and maximum OD for all sequenced strains used in the ML model under different growth conditions.

ASM does not own the copyrights to Supplemental Material that may be linked to, or accessed through, an article. The authors have granted ASM a non-exclusive, world-wide license to publish the Supplemental Material files. Please contact the corresponding author directly for reuse.
