# Supplementary material for: Single-strain behavior predicts responses to environmental pH and osmolality in the gut microbiota
Source: mBio. 2023 Jul 11;14(4):e00753-23. doi: 10.1128/mbio.00753-23 (PMC10470613; doi:10.1128/mbio.00753-23)
Supplement: Supplemental material — Figures S1 to S6 and captions for Tables S1 to S4. [file mbio.00753-23-s0001.docx]

**Supplementary Information**

Single-strain behavior predicts responses to environmental pH and osmolality in the gut microbiota

Katharine M. Ng^1,2*^, Sagar Pannu^1*^, Sijie Liu^2^, Juan C. Burckhardt^1^, Thad Hughes^3^, William Van Treuren^4^, Jen Nguyen^1^, Kisa Naqvi^2^, Bachviet Nguyen^1^, Charlotte A. Clayton^1^, Deanna M. Pepin^1^, Samuel R. Collins^1^, Carolina Tropini^1,2,5^

**Supplementary Figures**

**Figure S1. pH changes due to fermentation do not correlate with growth.** A) Maximum OD versus change in pH (measured with BCECF; Materials and Methods) under different conditions, labeled by bacterial family. B) Maximum OD versus maximum growth rate under different conditions, labeled by bacterial family.

**Figure S2. Osmolality (left) and pH (right) features show varying degrees of correlation.** Each square represents the number of strains for which the two thresholded features have the same value.

**
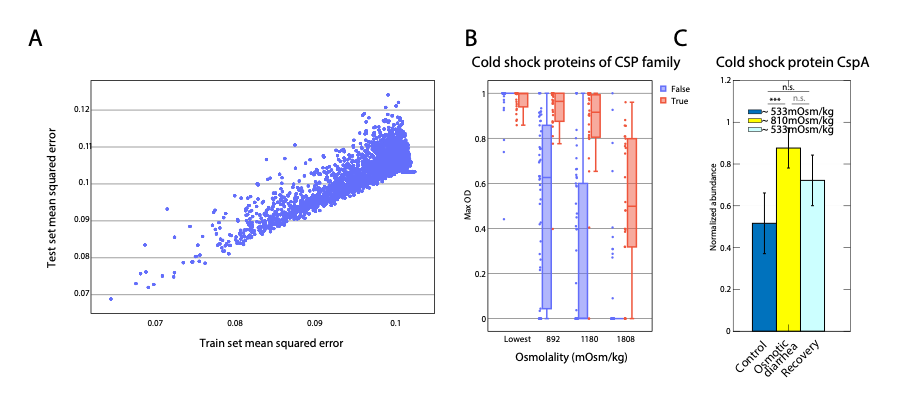
**

**Figure S3. Osmotic features identified by ML analysis are generalizable.** A) Measure of ML model generalization by comparing performance of model on training and held out test sets. Each point is a PATRIC feature for which we evaluated 5 K-fold models. B-C) Phenotypic variations in response to osmotic stress correlated with cold shock proteins: correlations occur both *in vitro* (B) and *in vivo* (C).

**Figure S4. Machine learning prediction errors for the pH dataset.** A) Most PATRIC features identified with ML predict that the presence of a specific feature will lead to a lower maximum normalized OD at pH 5.4. For features with a normalized maximum OD closer to 1, indicating good growth in acidic conditions, the features are heavily correlated with the family Lactobacillaceae (Materials and Methods). B) Measure of ML model generalization by comparing performance of model on training and held out test sets. Each point is a PATRIC feature for which we evaluated 5 K-fold models. C) PATRIC features do not correlate in their ability to predict growth in different osmolality and pH conditions. Total squared error for osmolality conditions versus total squared error for pH conditions.

**
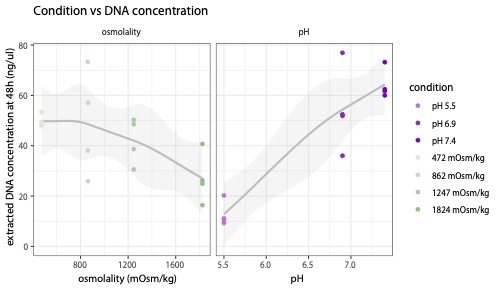
**

**Figure S5. DNA concentrations after 48h of growth of *in vitro* cultures of human fecal samples (n = 6) subjected to ranges of pH and osmolalities.**

**Figure S6. Taxa of interest in mice fed a control-diet or guar gum-supplemented diet.** Plot of relative abundance of taxa of interest across intestinal locations in mice fed different diets.

**Supplementary Table Legends**

**Table S1. Medium components for growth media used in the study.** Details are given for each strain, including the medium components, source, and sequencing results.

**Table S2. PATRIC features identified by ML to distinguish growth in different osmolality and pH conditions.** Columns indicate the name of the PATRIC feature, the threshold on the PATRIC count of the feature that distinguishes the phenotype, the number of strains whose count is lower than the threshold number of the feature, the number of strains whose count is higher than the threshold, and the total squared error for the mean of all ODs (Materials and Methods).

**Table S3. PATRIC features used in the ML model.** Each strain in our collection was annotated with PATRIC, and all features are represented in this table.

**Table S4. Normalized growth rate (GR) and maximum OD for all sequenced strains used in the ML model under different growth conditions.** Growth data outputs for each strain’s genome are combined with information obtained based on the *genome_name*.
